# Osteoradionecrosis after mandibular reconstruction: a comparative cohort study on quality of life and complications

**DOI:** 10.3389/fonc.2026.1758210

**Published:** 2026-02-04

**Authors:** Peng Zhang, Taiqing Lu, Ruiyan Gu, Wenli Yang

**Affiliations:** 1 Department of Prosthodontics, The First Affiliated Hospital of Zhengzhou University, Zhengzhou, China; 2 Department of Oral and Maxillofacial Surgery, The First Affiliated Hospital of Zhengzhou University, Zhengzhou, China; 3 Department of Stomatology, The First Affiliated Hospital of Zhengzhou University, Zhengzhou, China

**Keywords:** free fibula flap, mandibular reconstruction, osteoradionecrosis, quality of life, surgical complications

## Abstract

**Objective:**

This study aimed to compare longitudinal quality of life (QoL) and surgical outcomes following segmental mandibulectomy and free fibula flap reconstruction among patients with osteoradionecrosis (ORN), malignant disease, and benign conditions.

**Methods:**

A comparative cohort study was conducted involving 245 patients: 45 with ORN, 160 with malignancy, and 40 with benign disease. Patient-reported QoL was assessed using the EORTC QLQ-C30 and QLQ-H&N35 questionnaires preoperatively and at 3, 6, and 12 months postoperatively. The primary outcomes were 12-month Global Health Status (QLQ-C30) and Pain scores (QLQ-H&N35), analyzed using multivariable linear regression. The secondary outcome was the incidence of major surgical complications (Clavien-Dindo ≥ III), analyzed using multivariable logistic regression.

**Results:**

At 12 months, the ORN cohort demonstrated significantly worse QoL outcomes compared to the benign cohort, with a -14.1-point lower Global Health Status (95% CI: -19.5 to -8.7, p<0.001) and a +21.5-point higher Pain score (95% CI: +15.2 to +27.8, p<0.001), after multivariable adjustment. The ORN cohort also had the highest rate of major complications (48.9% vs. 17.5% benign, p<0.001), which remained significant in multivariable analysis (aOR for benign vs. ORN: 0.26, p=0.006). Larger bony defect length and longer operative time were independent predictors of poorer QoL and higher complication risk, while the use of virtual surgical planning was associated with better Global Health Status.

**Conclusion:**

Despite successful reconstruction, patients with ORN experience profoundly poorer long-term QoL, persistent pain, and a significantly higher complication burden compared to patients with benign or malignant disease. These findings highlight the unique challenges in ORN management and underscore the need for specialized long-term supportive care.

## Introduction

Segmental mandibulectomy followed by free fibula flap (FFF) reconstruction constitutes a cornerstone of contemporary head and neck reconstructive surgery, designed to restore both form and function in patients with substantial mandibular defects (1). This complex procedure is most frequently indicated following oncologic resections but also serves as a critical intervention for advanced osteoradionecrosis (ORN) which is a severe and debilitating complication of radiotherapy for head and neck cancer (2). ORN is characterized by progressive, non-healing, hypovascular bone necrosis, which often results in chronic pain, pathological fractures, orocutaneous fistula, and profound functional impairment. Although the technical success of microvascular reconstruction in this challenging patient population has been well established (3), a significant gap persists in our understanding of its long-term impact on patients’ quality of life (QoL) and overall recovery experience (4).

The existing literature underscores that patients with ORN present a uniquely complex clinical profile. As corroborated by the provided data, they are typically older, carry a higher burden of medical comorbidities, and exhibit surgical fields compromised by prior radiation therapy and multiple operations (5). These factors contribute to more extensive bony and soft tissue defects, prolonged operative durations, and inherently more complex reconstructions compared with patients undergoing reconstruction for benign conditions or primary malignancies (6). Consequently, it is widely accepted in clinical practice that patients with ORN face elevated rates of postoperative complications. Nevertheless, the subjective dimensions of their recovery including pain trajectories, functional rehabilitation, and overall well-being have been comparatively underexplored. Traditional surgical metrics, such as flap survival, while essential, fail to fully capture the persistent and often debilitating symptom burden that may define the daily lived experience of ORN survivors, even after technically successful reconstruction (7).

Accordingly, this study aims to provide a comprehensive, longitudinal comparison of QoL and surgical outcomes following mandibular reconstruction with FFF across three distinct cohorts: patients with ORN, those with malignant disease, and those with benign pathology. We hypothesize that, despite successful microvascular reconstruction, patients in the ORN cohort will demonstrate a significantly attenuated and incomplete QoL recovery trajectory relative to both control groups marked by persistent pain, enduring functional deficits, and a high symptom burden. Furthermore, we anticipate that the ORN cohort will exhibit a significantly greater incidence of major surgical complications, independent of other known risk factors.

## Methods

### Study design and patient population

This comparative cohort study, which involved a retrospective analysis of prospectively collected clinical data, was conducted at the First Affiliated Hospital of Zhengzhou University from January 2020 to October 2024. The study protocol received ethical approval from the hospital’s Institutional Review Board (No. CS-S202011), and written informed consent was obtained from all participants enrolled. Consecutive adult patients (≥ 18 years) scheduled to undergo segmental mandibulectomy with immediate FFF reconstruction were screened and stratified into three distinct cohorts based on their underlying diagnosis. The ORN Cohort comprised patients with a confirmed diagnosis of osteoradionecrosis, defined by a history of head and neck radiotherapy, persistent clinical symptoms (such as pain, exposed bone, or orocutaneous fistula), and radiological evidence of bone necrosis on computed tomography or magnetic resonance imaging. Prior conservative treatments for ORN, including hyperbaric oxygen therapy, prolonged antibiotic regimens, or local debridement, were documented but did not serve as exclusion criteria. The Malignant Control Cohort included patients undergoing resection for primary or recurrent malignancies involving the mandible with no cases of ORN included. The Benign Control Cohort consisted of patients with non-radiated, benign mandibular pathologies. Uniform exclusion criteria applied across all cohorts included: age under 18 years; inability to complete the quality of life questionnaires due to cognitive impairment, language barrier, or illiteracy; prior microvascular reconstruction to the same mandibular site; concurrent active malignancy at another anatomical site requiring treatment within 12 months; and severe, uncontrolled systemic comorbidities that would preclude safe surgical intervention or significantly confound the assessment of recovery. Patients who refused participation or were lost to follow-up before the 12-month assessment were also excluded to ensure data integrity for longitudinal analysis (**Supplementary Figure 1**).

### Sample size calculation

The sample size for this study was determined based on feasibility and availability of patients meeting the inclusion criteria during the study period (January 2020 to October 2024). Given the comparative cohort design and the relatively low incidence of advanced osteoradionecrosis requiring free fibula flap reconstruction, a formal power calculation was not performed *a priori*. Instead, we aimed to include all eligible consecutive patients from our institution to maximize statistical power and ensure representative data across the three cohorts.

### Data collection and variable

Data were collected through patient interviews, clinical examinations, and electronic medical records, encompassing a comprehensive set of variables. Baseline demographics and clinical variables included age, sex, body mass index (BMI), smoking and alcohol status, betel nut use, medical comorbidities, and detailed oncological history. Operative variables recorded were the extent of mandibular resection classified using the Boyd system, the dimensions of the bony and soft-tissue defect, operative and ischemia times, and specific reconstructive details including the number of fibula struts and the use of virtual surgical planning. The primary outcome variables were QOL assessed using the EORTC QLQ-C30 (version 3.0) and EORTC QLQ-H&N35 at preoperative, 3-month, 6-month, and 12-month intervals, and the incidence of major surgical complications defined as those requiring intervention (Clavien-Dindo Grade III or above). Secondary outcomes included flap success defined as complete survival without take-back for vascular compromise, and functional outcomes measured by interincisal distance and diet level, alongside other complications such as wound infection, fistula, hardware issues, and medical morbidities.

### Surgical technique

All procedures were performed by a single team of experienced microsurgeons to minimize technical variability. The surgical protocol involved aggressive resection of all necrotic bone and soft tissue in the ORN cohort until viable, bleeding margins were achieved. Mandibular reconstruction was performed using a free osteoseptocutaneous fibula flap as the first choice. The flap was inset and fixed using reconstruction plates, with osteotomies performed as needed. Microvascular anastomoses were performed to recipient vessels in the neck, typically the facial or superior thyroid arteries and veins.

### Statistical analysis

Descriptive statistics were presented as means with SD for normally distributed continuous variables, medians with interquartile ranges (IQR) for non-normal data, and frequencies with percentages for categorical variables. The analytical plan was structured to address the specific study aims. For the primary aim of comparing longitudinal QOL between the ORN and Benign Control cohorts, a linear mixed-effects model was employed. This model included fixed effects for cohort, time (as a categorical variable), and a cohort-by-time interaction term to test if the trajectory of QoL change differed between groups, while accounting for within-patient correlations across repeated measures. For the second primary aim, comparing the incidence of major surgical complications across all three cohorts, the Chi-square test was used for the omnibus test. If significant, *post-hoc* pairwise comparisons were performed using the Bonferroni correction to adjust for multiple comparisons. To control for potential confounding in both analyses, multivariable models were built: the linear mixed-model was adjusted for baseline covariates, and a multivariable logistic regression model was used for the complication outcome, with cohort assignment as the primary independent variable and the same set of confounders. A planned subgroup analysis compared outcomes based on mandibular defect location (anterior vs. lateral).

A *post-hoc* sensitivity analysis was conducted to examine the effect of prior radiotherapy within the malignant cohort. Patients in the malignant cohort were stratified into two subgroups: those who received adjuvant radiotherapy as part of their oncologic treatment (Malignant+RT) and those who did not (Malignant-RT). The 12-month QoL outcomes (Global Health Status from QLQ-C30 and Pain from QLQ-H&N35) were compared across four groups: Benign, Malignant-RT, Malignant+RT, and ORN, using multivariable linear regression adjusted for the same covariates as in the primary analysis.

All statistical analyses were performed using R 3.4.3, and a two-sided p-value of < 0.05 was considered statistically significant.

## Results

### Baseline data

A total of 245 patients were included finally, comprising 45 in the ORN cohort, 160 in the Malignant cohort, and 40 in the Benign cohort. The cohorts differed significantly in baseline profiles. Patients in the ORN cohort were older (mean age 62.5 years) and had a higher prevalence of risk factors, including current smoking (62.2%), alcohol use (48.9%), and betel nut use (17.8%), compared to the Benign cohort (all p<0.05). Medical comorbidities, particularly hypertension and diabetes mellitus, were also most common in the ORN cohort (84.4%, p<0.001). The ORN cohort was characterized by a history of prior radiation therapy (100%), with two-thirds (66.7%) also having undergone previous surgical intervention at the affected site. The distribution of mandibular defect location varied significantly, with the Benign cohort having the highest proportion of anterior defects (30.0%) and the Malignant cohort the lowest (13.8%, p=0.032) (**Table 1**).

**Table 1:** Baseline demographics and clinical characteristics.

Characteristic	ORN (n=45)	Malignant Cohort (n=160)	Benign Cohort (n=40)	p
Demographics				
Age, years, mean (SD)	62.5 (8.9)	59.1 (9.8)	41.3 (14.1)	**<0.001**
Sex, Male, n (%)	35 (77.8%)	112 (70.0%)	26 (65.0%)	0.381
BMI, kg/m², mean (SD)	22.1 (3.5)	23.1 (3.9)	24.8 (3.8)	**0.003**
Risk Factors				
Current Smoker, n (%)	28 (62.2%)	88 (55.0%)	6 (15.0%)	**<0.001**
Alcohol Use (>14 units/week), n (%)	22 (48.9%)	70 (43.8%)	5 (12.5%)	**<0.001**
Betel Nut Use, n (%)	8 (17.8%)	25 (15.6%)	0 (0%)	**0.016**
Medical Comorbidities				
Any Comorbidity, n (%)	38 (84.4%)	118 (73.8%)	16 (40.0%)	**<0.001**
• Hypertension	24 (53.3%)	72 (45.0%)	9 (22.5%)	**0.007**
• Diabetes Mellitus	12 (26.7%)	28 (17.5%)	3 (7.5%)	**0.035**
• Cardiovascular Disease	9 (20.0%)	22 (13.8%)	2 (5.0%)	0.103
• Chronic Pulmonary Disease	11 (24.4%)	38 (23.8%)	4 (10.0%)	0.131
Oncological History				
Prior Radiation, n (%)	45 (100%)	0 (0%)	0 (0%)	**<0.001**
Prior Radiation Dose, median (IQR)	66.0 (60.0 - 70.0)	–	–	–
Prior Surgery to Site, n (%)	30 (66.7%)	42 (26.3%)	8 (20.0%)	**<0.001**
Mandibular Defect Location				
Anterior, n (%)	10 (22.2%)	22 (13.8%)	12 (30.0%)	**0.032**
Lateral, n (%)	35 (77.8%)	138 (86.3%)	28 (70.0%)	**0.032**

BMI, Body Mass Index; Gy, Gray; IQR, Interquartile Range; SD, Standard Deviation. p-values are derived from ANOVA for continuous variables and Chi-square or Fisher's exact test for categorical variables. Significant p-values (<0.05) are in bold.

Significant differences in the complexity of resection and reconstruction were observed across the cohorts. The ORN cohort had the most extensive defects, with the longest median bony defect length (13.0 cm) and the largest median soft tissue defect area (45.0 cm², both p<0.05). Consequently, operative time was longest for the ORN cohort (median 585 minutes, p<0.001). Defect classification also differed, with a more complex distribution (Boyd Class H: ORN 22.2%, Benign 25.0%) compared to the Malignant cohort, which was predominantly Boyd Class L (73.7%, p<0.001). The use of VSP was highest in the Benign (82.5%) and ORN (71.1%) cohorts, compared to 51.3% in the Malignant cohort (p<0.001) (**Table 2**).

**Table 2:** Operative and reconstructive details.

Characteristic	ORN Cohort (n=45)	Malignant Cohort (n=160)	Benign Cohort (n=40)	p-value
Defect Classification (Boyd), n (%)				<0.001
Class A	10 (22.2%)	22 (13.8%)	12 (30.0%)	
Class L	25 (55.6%)	118 (73.7%)	18 (45.0%)	
Class H	10 (22.2%)	20 (12.5%)	10 (25.0%)	
Bony Defect Length, cm, median (IQR)	13.0 (11.0-15.0)	11.0 (9.0-13.0)	10.5 (8.0-12.0)	**<0.001**
Soft Tissue Defect Area, cm², median (IQR)	45.0 (35.0-60.0)	40.0 (30.0-54.5)	30.0 (24.0-45.0)	**0.002**
Operative Time, min, median (IQR)	585 (540-660)	510 (465-570)	545 (480-610)	**<0.001**
Ischemia Time, min, median (IQR)	95 (85-108)	92 (83-105)	90 (80-102)	0.215
Use of VSP, n (%)	32 (71.1%)	82 (51.3%)	33 (82.5%)	**<0.001**
Number of Fibula Struts, median (IQR)	2 (2-3)	2 (1-2)	3 (2-3)	**<0.001**

IQR, Interquartile Range; VSP, Virtual Surgical Planning. p-values are derived from Kruskal-Wallis test for continuous variables and Chi-square test for categorical variables. Significant p-values (<0.05) are in bold.

### QoL

**Figure 1:**
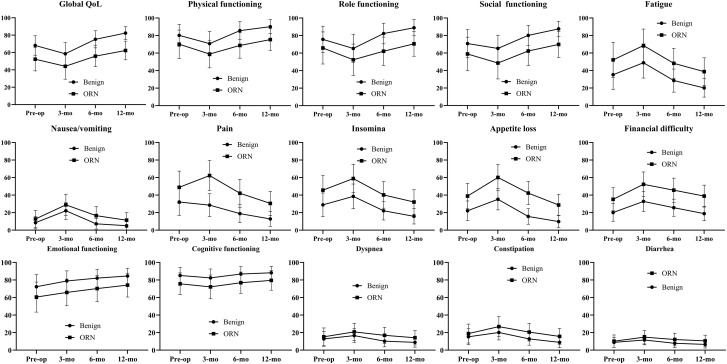
QLQ-C30 results in ORN and benign cohorts.

While all groups experienced the expected postoperative decline at 3 months, the ORN cohort’s scores failed to return to preoperative baselines in most domains by the 12-month mark. The ORN group’s Global Health Status, which started at a low preoperative level of 52.3, declined further to 44.1 at 3 months and recovered only to 62.3 at 12 months indicating a net improvement that still left patients with a poorer overall QoL than they had pre-surgery. Similarly, the Physical Functioning score of 75.4 at 12 months remained substantially higher that preoperative level of 70.1. This pattern of persistent deficit was even more pronounced in symptom scales. while showing some improvement over time, The ORN cohort reported severely elevated symptoms of Fatigue, Pain, and Insomnia at 3 months, which remained highly burdensome at 12 months. Symptoms like Appetite Loss were devastatingly high at 3 months (60.1) and, despite some improvement, remained severe at 12 months (28.7) (**Figure 1**; **Supplementary Table 1**).

**Figure 2:**
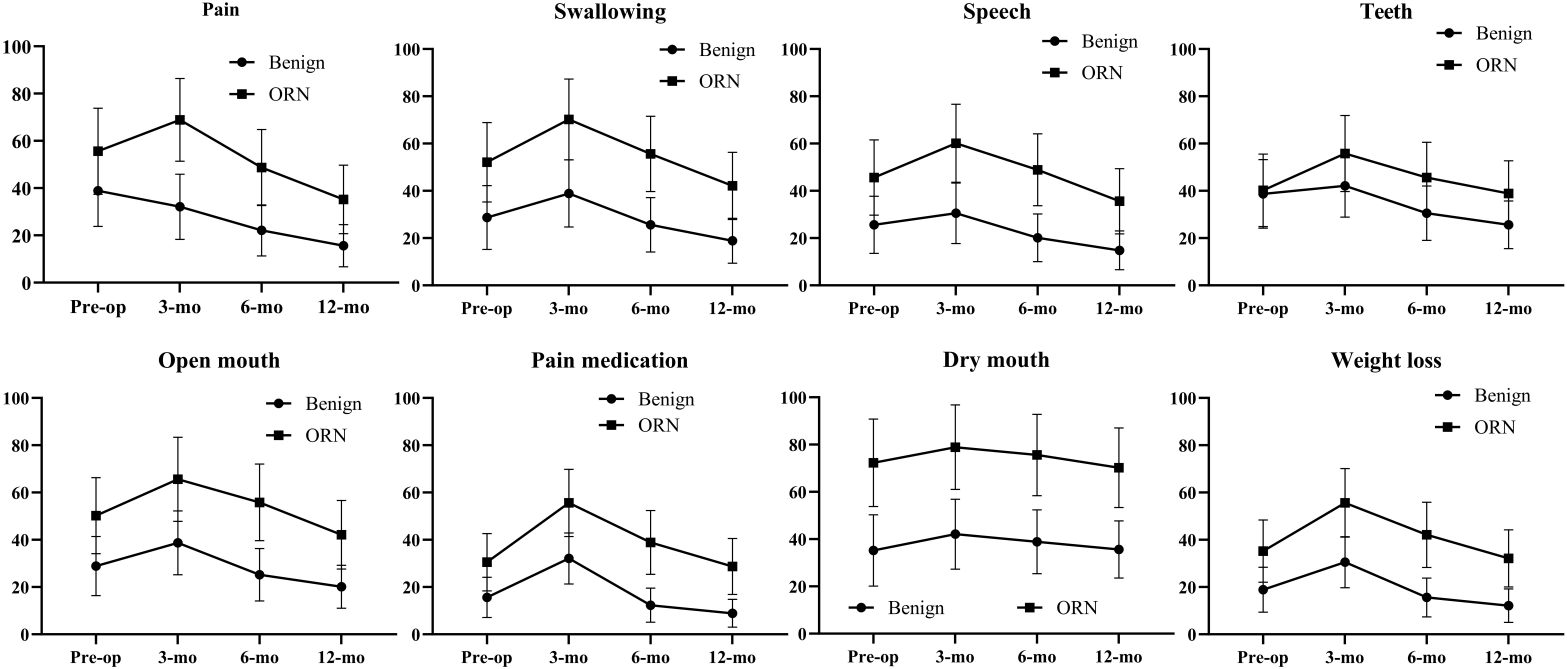
QLQ-H&N35 results in ORN and benign cohorts.

In the QLQ-H&N35, scores for Swallowing, Speech, Social Eating, and Social Contact showed only modest improvement from the severe postoperative peaks, remaining markedly elevated at 12 months. The legacy of prior radiotherapy was starkly evident in the persistently catastrophic scores for Dry Mouth and Sticky Saliva, which showed almost no improvement over the entire year. Furthermore, the ORN cohort’s continued reliance on substantial supportive care was reflected in persistently high use of Pain Medication, Nutritional Supplements, and Feeding Tubes at all postoperative intervals. For context, the Benign cohort demonstrated a robust “V-shaped” recovery, with scores not only rebounding but surpassing their preoperative baselines by 12 months. Consequently, the functional and symptomatic gap between the ORN and Benign cohorts remained large and statistically significant at all time points, underscoring the uniquely challenging and persistent burden of disease in ORN patients (**Figure 2**; **Supplementary Table 2**).

The selection of Global Health Status and Pain for the multivariable analysis was strategic. The global score provides a definitive, summative measure of overall patient well-being, demonstrating the comprehensive detriment of ORN on quality of life. The pain domain, a cardinal feature of ORN, pinpoints a key driver of this detriment. This dual approach assessing both overall burden and a specific, high-impact symptom robustly quantified the profound and independent negative effect of ORN, confirming it leads to both a significantly poorer quality of life and a severe, persistent pain syndrome. Belonging to the ORN cohort was associated with a 14.1-point lower Global Health Status (95% CI: -19.5 to -8.7, p<0.001) and a 21.5-point higher Pain score (95% CI: +15.2 to +27.8, p<0.001) at 12 months, compared to the Benign Control cohort. Surgical complexity and reconstruction details were significant independent contributors. Larger bony defect length was associated with poorer Global Health Status (β = -0.7 per cm, p=0.018) and higher pain (β = +0.8 per cm, p=0.025). The use of VSP was a protective factor, associated with a significantly better Global Health Status (β = +3.5, p=0.037). Longer operative time was an independent predictor of increased pain (β = +0.8 per 30 minutes, p=0.043). The presence of medical comorbidities also remained a significant predictor of higher pain (β = +5.2, p=0.046) (**Table 3**).

**Table 3:** Multivariable analysis of predictors for 12-month quality of life outcomes.

Predictor Variable	Outcome: Global Health Status / QoL (QLQ-C30)		Outcome: Pain Score (QLQ-H&N35)	
	β (95% CI)	p-value	β (95% CI)	p-value
Cohort (Ref: Benign)				
ORN Cohort	-14.1 (-19.5 to -8.7)	**<0.001**	+21.5 (+15.2 to +27.8)	**<0.001**
Age (per 1-year increase)	-0.1 (-0.3 to 0.1)	0.478	+0.1 (-0.2 to 0.4)	0.421
Sex (Ref: Female)				
Male	-1.2 (-5.0 to 2.6)	0.531	+2.5 (-1.9 to 6.9)	0.261
BMI (per 1 kg/m² increase)	+0.3 (0.0 to 0.6)	0.081	-0.3 (-0.7 to 0.1)	0.145
Current Smoker (Ref: No)	-2.9 (-6.9 to 1.1)	0.152	+4.1 (-0.8 to 9.0)	0.101
Any Comorbidity (Ref: No)	-3.8 (-8.0 to 0.4)	0.074	+5.2 (+0.1 to 10.3)	**0.046**
Defect Size (Ref: Boyd A)				
Boyd L	-3.1 (-7.9 to 1.7)	0.204	+4.2 (-1.3 to 9.7)	0.132
Boyd H	-6.5 (-12.2 to -0.8)	**0.026**	+8.9 (+2.3 to 15.5)	**0.009**
Bony Defect Length (per cm)	-0.7 (-1.3 to -0.1)	**0.018**	+0.8 (+0.1 to 1.5)	**0.025**
Operative Time (per 30-min)	-0.7 (-1.4 to 0.0)	0.051	+0.8 (0.0 to 1.6)	**0.043**
Use of VSP (Ref: No)	+3.5 (0.2 to 6.8)	**0.037**	-3.8 (-7.8 to 0.2)	0.063

β, Beta Coefficient; CI, Confidence Interval; Ref, Reference category; VSP, Virtual Surgical Planning. The model was adjusted for all variables listed. Significant p-values (<0.05) are in bold.

### Surgical complication

The incidence of major surgical complications differed significantly across the cohorts (p<0.001), with the ORN cohort experiencing the highest burden at 48.9% (22/45), compared to 25.0% (40/160) in the Malignant cohort and 17.5% (7/40) in the Benign cohort. Univariate analysis identified multiple factors associated with complication risk, including ORN cohort membership, smoking, comorbidities, prior surgery, larger defect size, and longer operative time. In the multivariable model, compared to the ORN cohort, the odds of a major complication were significantly lower in both the Malignant (aOR 0.41, 95% CI 0.21–0.81; p=0.010) and Benign (aOR 0.26, 95% CI 0.10–0.67; p=0.006) cohorts. Furthermore, larger bony defect length was a significant independent risk factor (aOR 1.20 per cm, 95% CI 1.06–1.35; p=0.003), while other variables, such as smoking and prior surgery, were attenuated and no longer significant in the adjusted model (**Table 4**).

**Table 4:** Univariate and multivariable analysis of predictors for major surgical complications.

Predictor variable	Univariate analysis		Multivariable analysis	
	OR [95% CI]	p-value	Adjusted OR [95% CI]	p-value
Cohort (Ref: ORN)				
Malignant Cohort	**0.36 [0.19 - 0.69]**	**0.002**	**0.41 [0.21 - 0.81]**	**0.010**
Benign Cohort	**0.22 [0.09 - 0.56]**	**0.001**	**0.26 [0.10 - 0.67]**	**0.006**
Age (per 1-year increase)	1.02 [0.99 - 1.05]	0.11	1.02 [0.99 - 1.05]	0.21
Sex (Ref: Female)				
Male	1.32 [0.76 - 2.28]	0.32	1.22 [0.70 - 2.14]	0.48
BMI (per 1 kg/m² increase)	1.01 [0.94 - 1.08]	0.75	1.01 [0.94 - 1.09]	0.82
Current Smoker (Ref: No)	**1.91 [1.11 - 3.28]**	**0.02**	1.50 [0.89 - 2.62]	0.13
Alcohol Use (Ref: No)	1.52 [0.89 - 2.59]	0.13	1.28 [0.72 - 2.25]	0.42
Betel Nut Use (Ref: No)	1.62 [0.88 - 2.98]	0.12	1.38 [0.72 - 2.65]	0.33
Any Comorbidity (Ref: No)	**2.12 [1.19 - 3.78]**	**0.01**	1.80 [0.99 - 3.27]	0.05
Prior Surgery to Site (Ref: No)	**2.29 [1.32 - 3.97]**	**0.003**	1.52 [0.80 - 2.87]	0.20
Defect Classification (Ref: Boyd A)				
Boyd L	1.72 [0.77 - 3.83]	0.18	1.40 [0.62 - 3.18]	0.42
Boyd H	**2.81 [1.11 - 7.10]**	**0.03**	1.92 [0.73 - 5.05]	0.19
Bony Defect Length (per cm)	**1.28 [1.15 - 1.43]**	**<0.001**	**1.20 [1.06 - 1.35]**	**0.003**
Soft Tissue Defect Area (per 10 cm²)	**1.22 [1.08 - 1.38]**	**0.001**	1.11 [0.98 - 1.26]	0.10
Operative Time (per 30-min)	**1.26 [1.10 - 1.44]**	**0.001**	1.12 [0.99 - 1.27]	0.07
Use of VSP (Ref: No)	0.71 [0.42 - 1.18]	0.18	0.80 [0.47 - 1.37]	0.42
Number of Fibula Struts (per strut)	1.35 [1.00 - 1.82]	0.05	1.22 [0.89 - 1.67]	0.22

CI, Confidence Interval; Ref, Reference category. Major complications defined as Clavien-Dindo Grade ≥ III. The multivariable logistic regression model was adjusted for all variables listed in the table. Significant p-values (<0.05) are in bold.

### Secondary outcome

Overall flap survival was high, with a 98.0% (240/245) success rate. There were five flap failures: three total losses (two in the ORN cohort, one in the Malignant cohort) and two partial losses requiring surgical revision (both in the ORN cohort). Consequently, the flap failure rate was significantly higher in the ORN cohort (8.9%, 4/45) compared to the Malignant (0.6%, 1/160) and Benign (0%, 0/40) cohorts (p<0.001).

Postoperative function was significantly impaired in the ORN cohort. The median interincisal distance at 12 months was 25.0 mm (IQR 18.0-30.0) in the ORN cohort, which was significantly lower than in the Malignant (35.0 mm, IQR 30.0-40.0; p<0.001) and Benign (42.0 mm, IQR 38.0-46.0; p<0.001) cohorts.

Dietary outcomes followed a similar pattern. At the 12-month follow-up, only 24.4% (11/45) of ORN patients had returned to a regular diet, compared to 55.0% (75/136) in the Malignant cohort and 87.5% (35/40) in the Benign cohort (p<0.001). A majority of ORN patients (57.8%, 26/45) remained on a soft or pureed diet.

### Mandibular defect location impact

Patients with anterior defects reported a significantly poorer Global Health Status (β = -5.8, 95% CI: -10.1 to -1.5, p=0.009) and higher Pain scores (β = +7.4, 95% CI: +2.1 to +12.7, p=0.006) at 12 months compared to those with lateral defects, after adjusting for cohort and other covariates. However, there was no significant interaction between defect location and cohort assignment for either QoL outcome (p-for-interaction > 0.10 for both), indicating that the detrimental effect of an anterior defect on quality of life was consistent across all three cohorts. Similarly, for major surgical complications, defect location was a significant independent predictor. Anterior defects were associated with higher odds of a major complication (aOR 2.15, 95% CI: 1.18 to 3.91, p=0.012) in the multivariable model. No significant interaction was found between cohort and defect location (p-for-interaction > 0.10), meaning that the increased risk associated with an anterior defect was uniform across the ORN, Malignant, and Benign cohorts.

### Impact of radiotherapy within the malignant cohort

After adjustment for covariates including age, defect size, operative time, and comorbidities, the Malignant+RT (n=112) subgroup exhibited significantly poorer QoL across nearly all domains compared to the Malignant-RT subgroup (n=48). Global QoL was 6.8 points lower (p=0.003), and Pain (QLQ-H&N35) was 9.4 points higher (p=0.002) in the Malignant+RT subgroup. QoL in the Malignant+RT subgroup closely paralleled that of the ORN cohort across multiple symptom domains, including fatigue, pain, dry mouth, and sticky saliva, underscoring the substantial and independent detriment of radiotherapy exposure on long-term patient-reported outcomes (**Supplementary Table 3**).

## Discussion

This comparative longitudinal study demonstrates that patients undergoing mandibular reconstruction for ORN endure a profoundly more challenging recovery trajectory than benign or malignant disease. Despite comparable technical success in reconstruction, the ORN cohort exhibited significantly poorer long-term QoL, characterized by a blunted recovery in global health status, severe and persistent pain, and enduring functional deficits across multiple domains. Furthermore, the ORN cohort experienced a substantially higher burden of major surgical complications independent of other risk factors. These findings collectively paint a sobering picture of ORN not merely as a surgical problem to be solved, but as a chronic, debilitating condition that leaves a lasting imprint on patient well-being, even after aggressive and successful microvascular reconstruction.

While the existing literature provides foundational insights into the management of mandibular ORN and its impact on QoL, much of this evidence is derived from retrospective cohort studies with inherent methodological limitations. Chang et al. (8) conducted a 10-year retrospective review of 35 patients with ORN, concluding that despite a 37% complication rate, surgical resection and reconstruction improved QoL. However, the study’s retrospective nature, small sample size, and limited use of a single modified QoL instrument restricted its generalizability and depth of patient-reported outcomes. Similarly, Jacobson et al. (9) performed a cross-sectional retrospective review of 42 ORN patients, assessing functional outcomes and QoL via telephone surveys and mailed questionnaires. Although they reported improved QoL post-surgery, the lack of preoperative baseline data and standardized longitudinal follow-up limited their ability to capture the true trajectory of recovery and the enduring burden of radiation-induced sequelae. Another retrospective study by Löfstrand et al. (10) compared QoL outcomes in 73 patients undergoing FFF reconstruction, including an ORN subgroup. While the authors identified poorer swallowing and social eating in ORN patients, the retrospective design and use of non-concurrent reference populations introduced potential recall and selection biases. Collectively, these retrospective studies underscore the challenges in ORN management but are constrained by the design lacking standardized preoperative assessments, and failing to capture dynamic recovery patterns (11).

To contextualize our findings within the broader evidence base, it is instructive to consider two recent systematic reviews that have synthesized the literature on QoL following FFF reconstruction for ORN. The 2022 systematic review by Tassone et al. (4) analyzed ten studies comprising 235 patients, the vast majority of whom had ORN. The authors concluded that while surgery reliably improved pain-related QoL, it did not ameliorate radiation-induced sequelae such as impaired chewing, swallowing, and salivary dysfunction, painting a picture of partial recovery dominated by enduring functional deficits. In parallel, the 2024 systematic review by Wu et al. (3), focusing on sequential fibula transfers, incorporated data from six articles reporting on 56 patients. This review highlighted that secondary reconstructions, often performed for ORN (39% of cases), were associated with a near-doubling of tube feeding dependence compared to primary procedures. However, it also noted that overall patient-reported QoL remained comparable to that after the first reconstruction, suggesting that patients adapt to a new normal. Collectively, these reviews underscore the persistent symptom burden and functional compromise associated with ORN reconstruction, while also hinting at a complex adaptation in patient-perceived well-being perceiving as a nuance our longitudinal, comparative study is uniquely positioned to explore and quantify.

Our findings not only corroborate the substantial disease burden of ORN reported in prior studies but further delineate a qualitatively distinct and impaired trajectory of recovery that underscores the profound clinical challenge these patients represent. The earlier prospective study by Danielsson et al. (12), while demonstrating post-reconstruction improvements in pain, emotional, and social functioning in a small ORN cohort, primarily established the potential for surgical benefit. In contrast, our larger, comparative longitudinal design reveals a critical nuance: ORN patients experience a markedly blunted and incomplete recovery pattern. Unlike the benign cohort, which exhibited a robust “V-shaped” rebound in QoL (returning to or exceeding preoperative baselines by 12 months) the ORN cohort failed to regain its own already-poorer preoperative status in most domains. This suggests that reconstruction for ORN, while ameliorating some severe symptoms, does not restore patients to a comparable recovery pathway enjoyed by those with non-irradiated defects. Furthermore, our results extend and refine the longitudinal framework proposed by Wu et al. (13), who noted a gradual QoL improvement post-reconstruction. Our data pinpoint the specific, persistent symptom drivers that sustain the QoL chasm between cohorts. ORN patients are plagued by enduringly high burdens of pain, fatigue, and insomnia long after surgery. Head and neck-specific functions such as swallowing, speech, and social eating remain severely impaired. Most strikingly, radiation-induced sequelae like dry mouth and sticky saliva show almost no improvement, highlighting the irreversible local tissue damage that microsurgery cannot address. This persistent multisymptom load captured serially over 12 months explains the stagnant global QoL scores and underscores that ORN is not merely a surgical defect but a chronic systemic condition. Thus, our study moves beyond confirming that ORN patients have worse outcomes; it elucidates how their recovery is fundamentally truncated and dominated by non-resolving symptoms, providing a more granular and sobering prognosis for patients and clinicians alike.

A significantly elevated major complication rate (48.9%) in ORN patients undergoing FFF reconstruction is observed comparing to benign and malignant cohorts. The high-risk profile of ORN reconstruction is a consistent theme across prior works. O’Connell et al. (14) reported a local wound complication rate of 47% and a 45% rate of hardware removal in their ORN cohort, markedly higher than in control groups. Baumann et al. (15), in their 10-year review, found an overall surgical complication rate of 32% and identified through-and-through defects as particularly challenging, with complication rates reaching 70% for bone flap reconstructions in type IIb defects. Fritz et al.’s contemporary review contextualizes this challenge, noting that traditional ORN management with segmental resection and osteocutaneous free flaps, while effective, is associated with high morbidity (16). The surgical difficulty is further underscored by technical reports: Kim et al. and Baron et al. both detailed the complex operative field in ORN, characterized by fibrosis, poor tissue quality, and scarce recipient vessels (17, 18). Liu et al. provided a predictive model, identifying specific risk factors such as radiotherapy interval ≤2 years and trismus as independent predictors of flap necrosis (19). Doub et al. (20) reported that in a cohort of 57 patients, 19 (33%) experienced a recurrence of infection. One- and two-year survival rates in this group were 75.8% and 66.2%, respectively. Crucially, the study identified nonviable resection margins as a significant predictor of earlier infection recurrence with an adjusted HR of 11.9 (95% CI: 3.84 to 36.7). While these studies collectively establish ORN as a high-risk surgical indication, our analysis introduces critical methodological and analytical novelty. First, prior studies were predominantly retrospective, single-cohort reviews or comparative analyses with limited adjustment for confounding variables. Our study, through a prospective, triple-cohort design (ORN vs. Malignant vs. Benign) with comprehensive multivariable adjustment, isolates the independent effect of the ORN disease entity itself. Second, whereas previous reports documented high absolute complication rates, our analysis quantifies the relative risk attributable to ORN. We demonstrate that, after controlling for defect size, operative time, and comorbidities, ORN patients retain nearly fourfold higher odds of a major complication compared to benign patients (aOR for benign vs. ORN: 0.26). This statistical confirmation that ORN is an independent risk factor, not merely a surrogate for larger defects or longer surgeries, represents a significant advancement. It underscores that the pathophysiological milieu of irradiated, hypovascular tissue itself is a primary driver of surgical morbidity, necessitating that risk stratification and patient counseling specifically account for the diagnosis of ORN as a key prognostic variable.

The impact of mandibular defect location on both QoL and surgical complications observed in our study aligns with and extends previous findings in the literature. Our results demonstrate that anterior defects are independently associated with significantly poorer Global Health Status, higher pain scores, and greater odds of major surgical complications. This finding is consistent with the anatomical and functional complexity of anterior mandibular reconstruction. The loss of critical muscle attachments in anterior defects can lead to impaired swallowing, speech articulation, and oral competence, as highlighted in recent long-term HRQoL studies (21). The lack of significant interaction between defect location and etiology underscores that the inherent challenges of anterior reconstruction, such as biomechanical instability, soft tissue deficits, and the need for complex rehabilitation, are universal, irrespective of the underlying pathology. This reinforces the notion that anterior mandibular defects represent a distinct and high-risk reconstructive category, necessitating meticulous planning, advanced techniques such as virtual surgical planning, and individualized rehabilitation protocols to mitigate their adverse impact on both functional outcomes and patient well-being.

Our *post-hoc* analysis further elucidates the role of radiotherapy in determining long-term QoL outcomes. Patients in the Malignant+RT subgroup exhibited significantly poorer QoL across multiple symptom domains compared to the Malignant-RT subgroup, with profiles that closely paralleled those of the ORN cohort in areas such as fatigue, pain, xerostomia, and sticky saliva. This observed hierarchy of impairment with the Benign cohort demonstrating the most favorable outcomes, followed by the Malignant-RT, Malignant+RT, and ORN cohorts in descending order suggests that radiotherapy exposure constitutes a graded risk for suboptimal QoL recovery, where ORN represents the most severe clinical endpoint of radiation-induced tissue damage. Importantly, these findings underscore that the negative impact on QoL is more attributable to the sequelae of radiation therapy than to the presence of malignancy, reinforcing that radiation-associated tissue injury fundamentally compromises the postoperative rehabilitative trajectory.

This study has several limitations that should be considered when interpreting its findings. First, while data were collected prospectively, the study design remains observational and non-randomized. Although we performed comprehensive multivariable adjustment for known confounders such as age, defect size, operative time, comorbidities, and prior radiation, residual confounding from unmeasured or unrecorded factors may persist. For example, variations in preoperative nutritional status, psychosocial support, and rehabilitation adherence were not systematically captured and could have differentially affected outcomes across cohorts. Second, the single-center nature of the study may affect the generalizability of the results. Surgical expertise, perioperative protocols, and patient demographics can vary across institutions, particularly in regions with differing healthcare resources or referral patterns. The high flap survival rate (98.0%) observed in our series may reflect the experience of our specialized microsurgical team and may not be replicable in all clinical settings. Third, the follow-up period was limited to 12 months, which may not fully capture the long-term trajectory of QoL, late complications, or adaptive changes in patients with osteoradionecrosis. ORN is a chronic condition, and symptoms such as pain, xerostomia, and functional impairment may evolve beyond the first postoperative year. Additionally, late complications such as hardware failure, recurrent infection, or secondary fibrosis may emerge after the study period, potentially underestimating the true long-term burden of the disease. Fourth, although we used validated instruments (EORTC QLQ-C30 and H&N35), patient-reported outcomes are inherently subjective and may be influenced by response bias, cultural factors, or varying expectations. The lack of a disease-specific module for osteoradionecrosis may also limit the sensitivity of our QoL assessment in capturing certain sequelae unique to irradiated tissue. Finally, while we stratified the malignant cohort by radiotherapy exposure, the ORN cohort itself was heterogeneous in terms of prior treatment history, radiation dose, and extent of necrosis. Subgroup analyses based on these factors were not performed due to sample size constraints, and thus the impact of specific clinical variables within the ORN population remains unclear. Future multi-center, longitudinal studies with longer follow-up, standardized rehabilitation pathways, and comprehensive capture of psychosocial and functional metrics are needed to validate and extend these findings. Incorporating objective functional assessments alongside patient-reported outcomes may provide a more holistic understanding of recovery in this complex patient population.

In summary, this comparative longitudinal study demonstrates that patients undergoing FFF reconstruction for ORN face a uniquely challenging recovery, marked by significantly poorer long-term QoL, persistent pain, and a higher burden of major complications compared to patients with benign or malignant mandibular disease. These findings underscore that ORN is not merely a surgical defect but a chronic, debilitating condition that continues to impair patient well-being even after technically successful reconstruction. The study highlights the critical need for a paradigm shift in ORN management that integrates meticulous surgical planning, such as virtual surgical planning, with dedicated, long-term multidisciplinary supportive care aimed at symptom management, functional rehabilitation, and psychosocial support. By recognizing ORN as an independent predictor of adverse outcomes, clinicians can better counsel patients, tailor interventions, and prioritize holistic care to mitigate the profound and lasting impact of this devastating condition.

## Data Availability

The original contributions presented in the study are included in the article/**Supplementary Material**. Further inquiries can be directed to the corresponding author.

## Supplementary material

Supplementary Figure 1 Flowchart illustrating patient screening, exclusion, and cohort formation.
