# Supplementary figures and images for: Osteoradionecrosis after mandibular reconstruction: a comparative cohort study on quality of life and complications
Source: Front Oncol. 2026 Feb 4;16:1758210. doi: 10.3389/fonc.2026.1758210 (PMC12913075; doi:10.3389/fonc.2026.1758210)

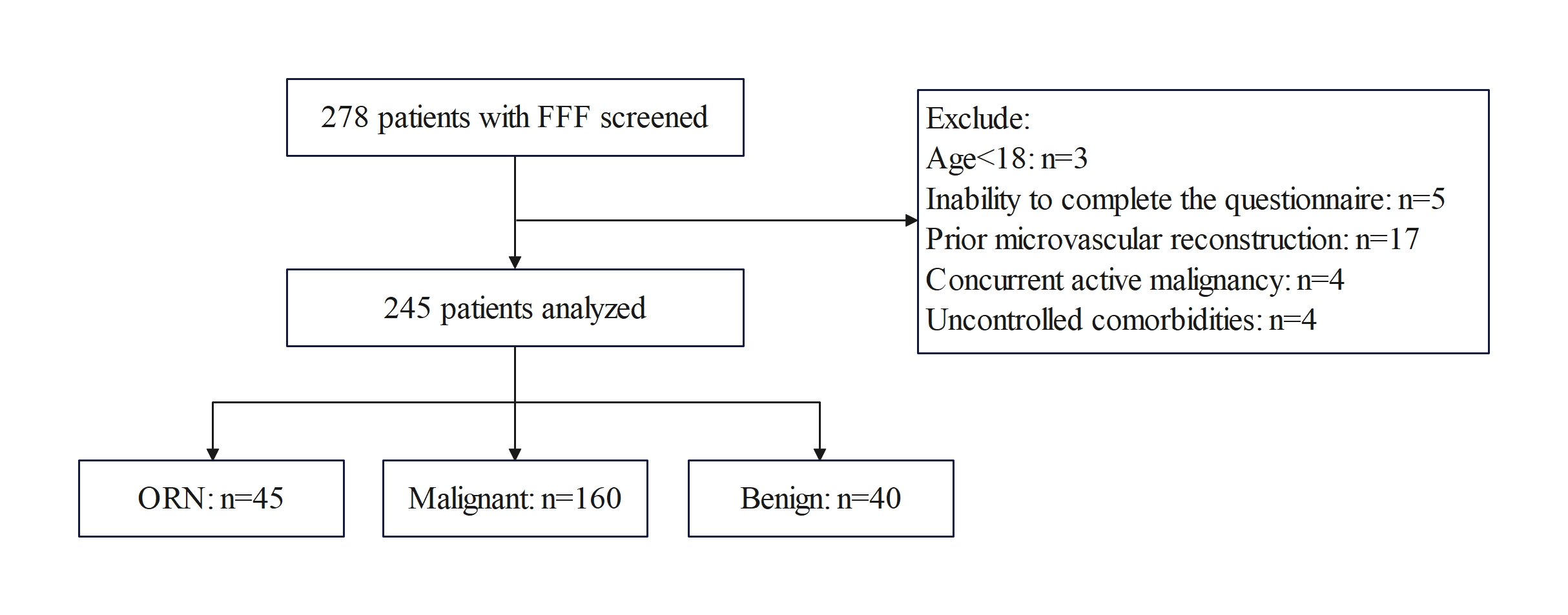

Supplement: Supplementary Figure 1 — Flowchart illustrating patient screening, exclusion, and cohort formation. [file Image1.jpeg]
