# Supplementary material for: Osteoradionecrosis after mandibular reconstruction: a comparative cohort study on quality of life and complications
Source: Front Oncol. 2026 Feb 4;16:1758210. doi: 10.3389/fonc.2026.1758210 (PMC12913075; doi:10.3389/fonc.2026.1758210)
Supplement: Supplementary file 2 [file Table1.doc]

### ****Supplementary Table 1: Longitudinal Health-Related Quality of Life (EORTC QLQ-C30)****

| **Domain** | Preop Benign | Preop ORN | 3mo Benign | 3mo ORN | 6mo Benign | 6mo ORN | 12mo Benign | 12mo ORN |
| --- | --- | --- | --- | --- | --- | --- | --- | --- |
| ****Global QoL**** | 68.1 (11.2) | 52.3 (13.5) | 58.5 (12.8) | 44.1 (14.9) | ****75.2 (9.8)**** | 55.8 (12.1) | ****82.4 (7.5)**** | 62.3 (10.8) |
| ****Physical functioning**** | 80.2 (12.5) | 70.1 (16.2) | 70.8 (14.1) | 58.9 (15.8) | ****85.6 (10.2)**** | 68.7 (14.5) | ****90.1 (8.1)**** | 75.4 (12.3) |
| ****Role functioning**** | 75.6 (15.1) | 65.8 (18.3) | 65.3 (16.2) | 52.4 (17.9) | ****82.3 (11.8)**** | 62.1 (16.2) | ****88.9 (9.2)**** | 70.5 (14.1) |
| ****Emotional functioning**** | 72.3 (13.8) | 60.5 (17.1) | 78.9 (11.5) | 65.8 (15.2) | 82.1 (10.1) | 70.2 (14.8) | 84.5 (8.9) | 74.1 (13.6) |
| ****Cognitive functioning**** | 85.1 (9.2) | 75.6 (12.4) | 82.4 (10.1) | 72.1 (13.5) | 86.8 (8.5) | 76.9 (12.1) | 88.2 (7.3) | 79.5 (11.2) |
| ****Social functioning**** | 70.8 (16.1) | 58.9 (19.2) | ****65.2 (15.2)**** | 48.5 (18.1) | ****80.1 (11.5)**** | 62.3 (16.8) | ****87.5 (8.8)**** | 69.8 (14.9) |
| ****Fatigue**** | 35.2 (16.8) | 52.1 (20.1) | 48.9 (17.5) | ****68.5 (18.9)**** | ****28.5 (13.2)**** | 48.2 (17.1) | ****20.1 (10.5)**** | 38.7 (15.8) |
| ****Nausea/vomiting**** | 8.5 (7.1) | 12.8 (9.8) | 22.1 (9.8) | 28.9 (12.1) | ****7.2 (6.0)**** | 16.5 (10.2) | 5.1 (4.8) | 11.4 (8.5) |
| ****Pain**** | 32.1 (15.2) | 48.9 (18.5) | ****28.5 (13.1)**** | ****62.3 (17.2)**** | ****18.9 (10.2)**** | 42.1 (15.9) | ****12.8 (8.5)**** | 30.5 (13.8) |
| ****Dyspnea**** | 12.8 (8.5) | 15.2 (10.1) | 16.5 (7.9) | 20.8 (9.8) | 10.2 (6.5) | 16.9 (9.1) | 8.9 (5.5) | 14.1 (8.2) |
| ****Insomnia**** | 28.9 (13.5) | 45.6 (16.8) | 38.5 (14.1) | ****58.9 (16.2)**** | ****22.1 (10.5)**** | 40.2 (15.1) | ****15.8 (8.8)**** | 32.1 (13.9) |
| ****Appetite loss**** | 22.1 (11.2) | 38.9 (14.5) | ****35.2 (12.1)**** | ****60.1 (14.8)**** | ****15.6 (8.9)**** | 42.3 (13.2) | ****9.8 (7.1)**** | 28.7 (12.1) |
| ****Constipation**** | 15.2 (9.1) | 18.9 (10.8) | 20.1 (8.5) | 26.8 (11.5) | 12.8 (7.2) | 20.5 (10.1) | 8.9 (6.1) | 15.6 (9.2) |
| ****Diarrhea**** | 8.9 (5.8) | 10.2 (7.1) | 11.5 (5.5) | 14.8 (7.8) | 7.8 (4.9) | 12.1 (7.2) | 6.5 (4.1) | 10.5 (6.5) |
| ****Financial difficulty**** | 20.1 (10.1) | 35.2 (13.2) | 32.8 (11.5) | ****52.3 (14.1)**** | 25.6 (9.8) | 45.6 (13.8) | ****18.9 (8.2)**** | 38.9 (12.5) |

*Scoring: Functioning/QoL scales: higher score = better. Symptom scales: higher score = worse. Bold indicates significant difference (p<0.05) between cohorts at that time point. Data are mean (SD).*
