# Supplementary material for: Osteoradionecrosis after mandibular reconstruction: a comparative cohort study on quality of life and complications
Source: Front Oncol. 2026 Feb 4;16:1758210. doi: 10.3389/fonc.2026.1758210 (PMC12913075; doi:10.3389/fonc.2026.1758210)
Supplement: Supplementary file 3 [file Table2.doc]

### ****Supplementary Table 2: Longitudinal Head and Neck-Specific Quality of Life (EORTC QLQ-H&N35)****

| **Domain** | **Preop Benign** | **Preop ORN** | **3mo Benign** | **3mo ORN** | **6mo Benign** | **6mo ORN** | **12mo Benign** | **12mo ORN** |
| --- | --- | --- | --- | --- | --- | --- | --- | --- |
| ****Pain**** | 38.9 (15.1) | 55.6 (18.2) | ****32.1 (13.8)**** | ****68.9 (17.5)**** | ****22.1 (10.8)**** | 48.7 (16.1) | ****15.6 (8.9)**** | 35.2 (14.5) |
| ****Swallowing**** | 28.7 (13.5) | 52.1 (16.8) | ****38.9 (14.2)**** | ****70.2 (17.1)**** | ****25.6 (11.5)**** | 55.6 (15.9) | ****18.9 (9.5)**** | 42.1 (14.2) |
| ****Senses**** | 20.1 (10.8) | 35.2 (14.1) | 25.6 (11.5) | 42.1 (15.2) | 18.9 (9.1) | 38.9 (14.5) | 15.2 (7.8) | 32.1 (13.1) |
| ****Speech**** | 25.6 (12.1) | 45.6 (15.9) | ****30.5 (12.8)**** | ****60.1 (16.5)**** | ****20.1 (10.1)**** | 48.9 (15.2) | ****14.8 (8.2)**** | 35.6 (13.8) |
| ****Social eating**** | 32.1 (14.2) | 58.9 (17.5) | ****42.1 (15.1)**** | ****75.6 (18.2)**** | ****28.9 (12.1)**** | 62.3 (16.8) | ****20.5 (9.8)**** | 48.7 (15.1) |
| ****Social contact**** | 22.1 (10.5) | 42.3 (14.8) | ****28.9 (11.2)**** | ****58.7 (16.1)**** | ****18.9 (9.1)**** | 50.2 (15.2) | ****14.1 (7.5)**** | 38.9 (13.5) |
| ****Sexual problems**** | 45.6 (17.1) | 60.2 (19.8) | 48.9 (16.2) | 65.8 (19.1) | 42.1 (15.1) | 58.9 (18.5) | 38.9 (13.8) | 55.6 (17.9) |
| ****Teeth**** | 30.5 (13.2) | 52.3 (16.5) | ****38.9 (14.1)**** | ****65.8 (17.2)**** | ****25.6 (11.8)**** | 58.7 (16.1) | ****22.1 (9.8)**** | 48.9 (14.9) |
| ****Open mouth**** | 28.9 (12.5) | 50.2 (16.1) | ****38.7 (13.5)**** | ****65.6 (17.8)**** | ****25.2 (11.1)**** | 55.8 (16.2) | ****20.1 (9.1)**** | 42.1 (14.5) |
| ****Dry mouth**** | 35.2 (15.1) | 72.3 (18.5) | 42.1 (14.8) | ****78.9 (17.9)**** | 38.9 (13.5) | ****75.6 (17.2)**** | 35.6 (12.1) | ****70.2 (16.8)**** |
| ****Sticky saliva**** | 30.5 (13.8) | 68.9 (17.2) | 38.7 (14.2) | ****75.2 (17.5)**** | 32.1 (12.5) | ****70.8 (16.9)**** | 28.9 (11.1) | ****65.6 (16.1)**** |
| ****Cough**** | 22.1 (10.5) | 35.6 (14.1) | 28.9 (11.2) | 42.1 (15.2) | 20.1 (9.1) | 38.9 (14.5) | 18.9 (8.2) | 32.1 (13.1) |
| ****Feeling ill**** | 25.6 (12.1) | 45.2 (15.8) | 32.1 (12.8) | 55.6 (16.5) | 22.1 (10.5) | 48.7 (15.9) | 18.9 (8.9) | 40.2 (14.2) |
| ****Pain medication**** | 15.6 (8.5) | 30.5 (12.1) | ****32.1 (10.8)**** | ****55.6 (14.2)**** | ****12.3 (7.2)**** | 38.9 (13.5) | ****8.9 (5.9)**** | 28.7 (11.8) |
| ****Food supplement**** | 12.1 (7.2) | 25.6 (11.5) | ****28.9 (9.8)**** | ****52.3 (13.8)**** | ****10.2 (6.5)**** | 35.2 (12.1) | ****6.5 (4.8)**** | 22.1 (10.5) |
| ****Feeding tube**** | 8.9 (5.9) | 20.1 (9.8) | ****22.1 (8.5)**** | ****48.7 (12.9)**** | ****7.8 (5.1)**** | 30.5 (11.2) | ****4.8 (3.9)**** | 18.9 (9.5) |
| ****Weight loss**** | 18.9 (9.5) | 35.2 (13.2) | ****30.5 (10.8)**** | ****55.6 (14.5)**** | ****15.6 (8.2)**** | 42.1 (13.8) | ****12.1 (7.1)**** | 32.1 (12.1) |
| ****Weight gain**** | 15.6 (6.5) | 10.2 (7.8) | 12.1 (5.9) | 8.9 (6.8) | 20.1 (7.2) | 12.3 (8.1) | 22.1 (8.5) | 15.6 (9.2) |

*Scoring: All scales are symptom scales; higher score = worse symptom burden. Bold indicates significant difference (p<0.05) between cohorts at that time point. Data are mean (SD).*
