# Supplementary material for: Osteoradionecrosis after mandibular reconstruction: a comparative cohort study on quality of life and complications
Source: Front Oncol. 2026 Feb 4;16:1758210. doi: 10.3389/fonc.2026.1758210 (PMC12913075; doi:10.3389/fonc.2026.1758210)
Supplement: Supplementary file 4 [file Table3.doc]

### ****Supplementary Table 3: Adjusted 12-Month QoL Scores Stratified by Radiotherapy Status in the Malignant Cohort****

| ****Domain**** | ****Benign****  ****(n=40)**** | ****Malignant-RT (n=48)**** | ****Malignant+RT (n=112)**** | ****ORN****  ****(n=45)**** |
| --- | --- | --- | --- | --- |
| ****QLQ-C30 Scales**** |  |  |  |  |
| ****Global QoL**** | 82.4 (7.5) | 74.2 (10.1)* | 67.4 (12.3)*† | 62.3 (10.8)*† |
| ****Physical functioning**** | 90.1 (8.1) | 82.3 (11.2)* | 76.5 (13.8)*† | 75.4 (12.3)*† |
| ****Role functioning**** | 88.9 (9.2) | 80.1 (13.5)* | 72.8 (15.1)*† | 70.5 (14.1)*† |
| ****Emotional functioning**** | 84.5 (8.9) | 78.9 (11.8) | 73.2 (14.5)*† | 74.1 (13.6)* |
| ****Cognitive functioning**** | 88.2 (7.3) | 82.1 (10.5) | 78.5 (12.8)*† | 79.5 (11.2)*† |
| ****Social functioning**** | 87.5 (8.8) | 78.9 (12.1)* | 71.2 (15.2)*† | 69.8 (14.9)*† |
| ****Fatigue**** | 20.1 (10.5) | 28.9 (14.2)* | 38.5 (16.8)*† | 38.7 (15.8)*† |
| ****Nausea/vomiting**** | 5.1 (4.8) | 8.9 (7.2) | 12.3 (9.5)*† | 11.4 (8.5)*† |
| ****Pain**** | 12.8 (8.5) | 22.1 (12.1)* | 30.8 (15.2)*† | 30.5 (13.8)*† |
| ****Dyspnea**** | 8.9 (5.5) | 12.3 (8.1) | 16.8 (10.2)*† | 14.1 (8.2)* |
| ****Insomnia**** | 15.8 (8.8) | 25.6 (12.1)* | 35.2 (15.8)*† | 32.1 (13.9)*† |
| ****Appetite loss**** | 9.8 (7.1) | 18.9 (11.2)* | 28.5 (14.8)*† | 28.7 (12.1)*† |
| ****Constipation**** | 8.9 (6.1) | 12.1 (8.5) | 18.9 (11.5)*† | 15.6 (9.2)*† |
| ****Diarrhea**** | 6.5 (4.1) | 9.8 (6.5) | 12.1 (8.9)*† | 10.5 (6.5)* |
| ****Financial difficulty**** | 18.9 (8.2) | 25.6 (11.5)* | 38.9 (15.2)*† | 38.9 (12.5)*† |
| ****QLQ-H&N35 Scales**** |  |  |  |  |
| ****Pain**** | 15.6 (8.9) | 26.8 (13.2)* | 36.2 (16.5)*† | 35.2 (14.5)*† |
| ****Swallowing**** | 18.9 (9.5) | 28.5 (13.8)* | 40.1 (17.2)*† | 42.1 (14.2)*† |
| ****Senses**** | 15.2 (7.8) | 22.1 (11.5) | 30.8 (15.2)*† | 32.1 (13.1)*† |
| ****Speech**** | 14.8 (8.2) | 25.6 (12.1)* | 35.2 (16.5)*† | 35.6 (13.8)*† |
| ****Social eating**** | 20.5 (9.8) | 32.1 (14.1)* | 45.6 (18.5)*† | 48.7 (15.1)*† |
| ****Social contact**** | 14.1 (7.5) | 25.2 (12.5)* | 38.9 (16.8)*† | 38.9 (13.5)*† |
| ****Sexual problems**** | 38.9 (13.8) | 45.6 (16.2) | 52.3 (18.9)*† | 55.6 (17.9)*† |
| ****Teeth**** | 22.1 (9.8) | 32.1 (13.5)* | 45.6 (17.8)*† | 48.9 (14.9)*† |
| ****Open mouth**** | 20.1 (9.1) | 30.5 (13.2)* | 42.1 (17.5)*† | 42.1 (14.5)*† |
| ****Dry mouth**** | 35.6 (12.1) | 48.9 (15.2)* | 65.8 (18.1)*† | 70.2 (16.8)*† |
| ****Sticky saliva**** | 28.9 (11.1) | 38.9 (14.2)* | 58.7 (18.5)*† | 65.6 (16.1)*† |
| ****Cough**** | 18.9 (8.2) | 25.2 (11.5) | 32.1 (15.2)*† | 32.1 (13.1)*† |
| ****Feeling ill**** | 18.9 (8.9) | 28.9 (13.2)* | 38.9 (16.8)*† | 40.2 (14.2)*† |
| ****Pain medication**** | 8.9 (5.9) | 18.9 (10.8)* | 28.9 (14.2)*† | 28.7 (11.8)*† |
| ****Food supplement**** | 6.5 (4.8) | 15.6 (9.8)* | 25.2 (13.8)*† | 22.1 (10.5)*† |
| ****Feeding tube**** | 4.8 (3.9) | 12.3 (8.5)* | 22.1 (12.1)*† | 18.9 (9.5)*† |
| ****Weight loss**** | 12.1 (7.1) | 22.1 (11.2)* | 32.1 (15.2)*† | 32.1 (12.1)*† |
| ****Weight gain**** | 22.1 (8.5) | 18.9 (10.2) | 15.6 (11.5)*† | 15.6 (9.2)*† |

****Scoring:**** QLQ-C30 Functioning/QoL scales: higher score = better. QLQ-C30 & QLQ-H&N35 Symptom scales: higher score = worse.
****Data presented as:**** Adjusted mean (SD) from multivariable linear regression.

p<0.05 vs. Benign cohort
† p<0.05 vs. Malignant-RT subgroup
